# Syntheses, crystal structures and Hirshfeld surface analyses of *N*-aryl­sulfonyl derivatives of cytisine

**DOI:** 10.1107/S2056989023001950

**Published:** 2023-03-10

**Authors:** Rasul Ya. Okmanov, Manzura I. Olimova, Surayyo B. Karabaeva, Frunza A. Sapaev, Kudaybergen B. Abdireymov

**Affiliations:** a S. Yunusov Institute of the Chemistry of Plant Substances, Academy of Sciences of Uzbekistan, Mirzo Ulugbek Str. 77, Tashkent, 100170, Uzbekistan; b National University of Uzbekistan named after Mirzo Ulugbek, massif Universitet shakharchasi 4, Tashkent, 100174, Uzbekistan; c Kara-Kalpak State University, acad. Abdirov Str., 1, Nukus, 742000, Uzbekistan; Vienna University of Technology, Austria

**Keywords:** alkaloid, cytisine, aryl­sulfonyl­ation, synthesis, crystal structure, Hirshfeld surface analysis

## Abstract

By aryl­sulfonyl­ation of cytisine in the presence of tri­ethyl­amine, three new compounds have been obtained in good yields: (7*R*,9*R*)-*N*-[(4-ethyl­phen­yl)sulfon­yl]cytisine, C_19_H_22_N_2_O_3_S (**I**) {systematic name: (1*R*,5*R*)-3-[(4-ethyl­phen­yl)sulfon­yl]-1,2,3,4,5,6-hexa­hydro-8*H*-1,5-methano­pyrido[1,2-*a*][1,5]diazo­cin-8-one}, (7*R*,9*R*)-*N*-[(4-chloro­phen­yl)sulfon­yl]cytisine, C_17_H_17_ClN_2_O_3_S (**II**) {systematic name: (1*R*,5*R*)-3-[(4-chloro­phen­yl)sulfon­yl]-1,2,3,4,5,6-hexa­hydro-8*H*-1,5-methano­pyrido[1,2-*a*][1,5]diazo­cin-8-one} and (7*R*,9*R*)-*N*-[(3-nitro­phen­yl)sulfon­yl]cytisine, C_17_H_17_N_3_O_5_S (**III**) {systematic name: (1*R*,5*R*)-3-[(3-nitro­phen­yl)sulfon­yl]-1,2,3,4,5,6-hexa­hydro-8*H*-1,5-methano­pyrido[1,2-*a*][1,5]diazo­cin-8-one}. The crystal structures of the compounds were determined on the basis of single-crystal X-ray diffraction data. The crystal structures of (**I**)–(**III**) are distinguished by the arrangement of two fragments of the mol­ecule around the sulfonyl site. For all structures, weak C—H⋯O hydrogen bonds are developed. Hirshfeld surface analysis shows that H⋯H (for **I** and **II**) and H⋯O/O⋯H (for **III**) inter­actions make the most important contribution to the crystal packing.

## Abstract 2

Aryl­sulfonation of cytisine with three types of substituted aryl­sulfonyl chlorides produced products (**I**)–(**III**), the mol­ecular structures of which differ in the location of the benzene fragment relative to the cytisine core. Inter­molecular C—H⋯O hydrogen bonds cross-link the mol­ecules into infinite chains.

## 1. Chemical context

Cytisine was first isolated by Huzeman and Marme from the seeds of *Cytisus Laburnum* Med. in 1865. To this day, other sources of cytisine have been found (Azimova & Yunusov, 2013 ▸), mainly isolated from plants of the legume family (especially the seeds of *Laburnum anagyroides*). Cytisine is a quinolizidine alkaloid, which is found in different sources by different names: 1,2,3,4,5,6-hexa­hydro-1,5-methano-8*H*-pyrido(1,2-*a*)(1,5)-diazo­cin-8-one (Freer *et al.*, 1987 ▸), 7,11-di­aza­tri­cyclo­[7.3.1.0^2,7^]trideca-2,4-dien-6-one (Kulakov *et al.*, 2010 ▸), (1*R*,5*S*)-cytisine (Rouden *et al.*, 2014 ▸) or (7*R*,9*S*)-cytisine.

Various studies report modern methods for the synthesis of cytisine (Barát *et al.*, 2018 ▸; Hirschhäuser *et al.*, 2011 ▸; O’Neill *et al.*, 2000 ▸; Pérez *et al.*, 2012 ▸) or cytisine modification (Brel, 2016 ▸; Kulakov *et al.*, 2010 ▸; Kulakov & Nurkenov, 2012 ▸; Shishkin *et al.*, 2010 ▸; Marrière *et al.*, 2000 ▸; Frasinyuk *et al.*, 2007 ▸). From the large number of cytisine derivatives, substances with biological activity (Tutka *et al.*, 2019 ▸; Gotti & Clementi, 2021 ▸; Liu *et al.*, 2020 ▸) and agents used in medicine (Tabex) have been found. From a chemical point of view, derivation studies of cytisine as well as the development of new methods for the synthesis of various cytisine derivatives are of inter­est. 
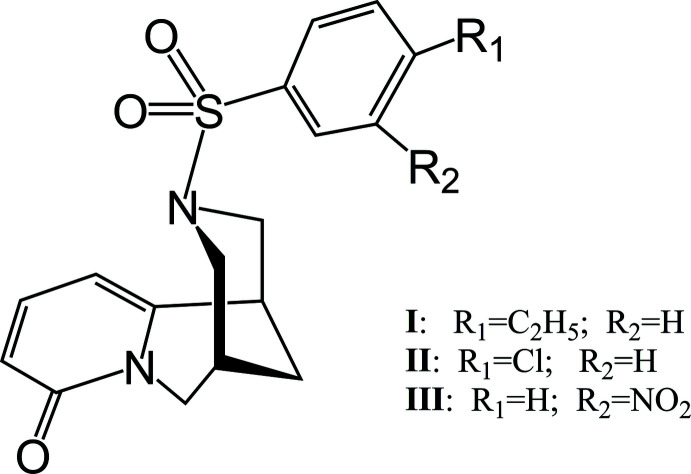


This communication describes the synthesis and crystal structures of three *N*-aryl­sulfonyl derivatives of cytisine. To obtain these *N*-aryl­sulfonyl derivatives, 4-ethyl­benzene­sulfonyl chloride, 4-chloro­benzene­sulfonyl chloride and 3-nitro­benzene­sulfonyl chloride were used, resulting in (7*R*,9*R*)-*N*-[(4-ethyl­phen­yl)sulfon­yl]cytisine (**I**), (7*R*,9*R*)-*N*-[(4-chloro­phen­yl)sulfon­yl]cytisine (**II**) and (7*R*,9*R*)-*N*-[(3-nitro­phen­yl)sulfon­yl]cytisine (**III**).

## 2. Structural commentary

The conformations of the cytisine cores in structures (**I**)–(**III**) are virtually identical and also do not differ from that of the cytisine mol­ecule itself (Freer *et al.*, 1987 ▸), or its various *N*-derivatives. The configurations of the chiral C atoms in cytisine are 7*R*, 9*S*, whereas in the case of (**I**)–(**III**) obtained by aryl­sulfonation of cytisine, the configurations are 7*R*, 9*R* in each case.

**Figure 1:**
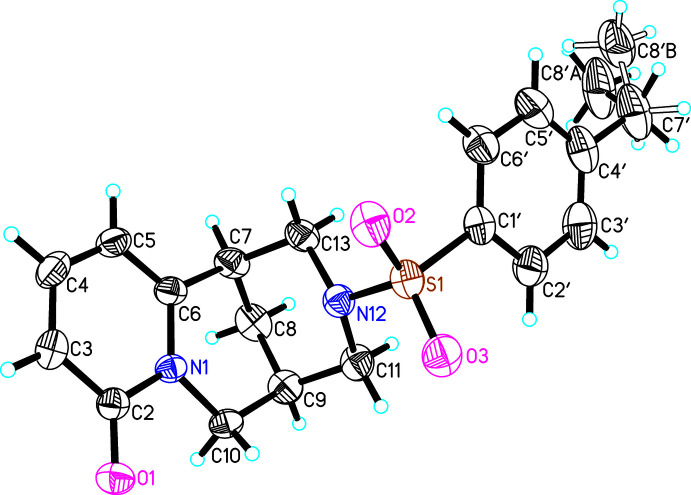
The asymmetric unit of (**I**) with atom labelling. Displacement ellipsoids represent 30% probability levels.

**Figure 2:**
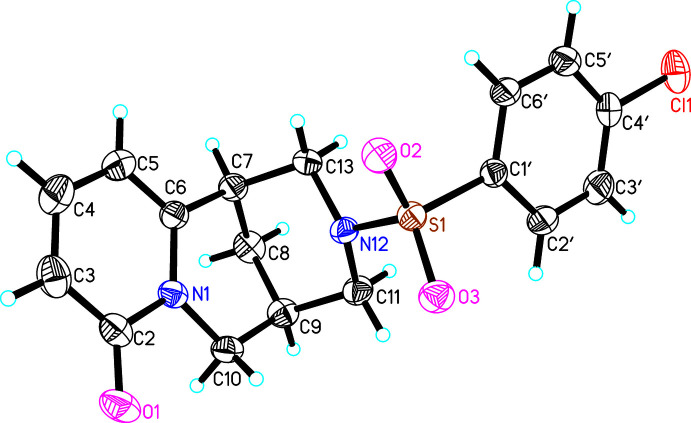
The asymmetric unit of (**II**) with atom labelling. Displacement ellipsoids represent 30% probability levels.

**Figure 4:**
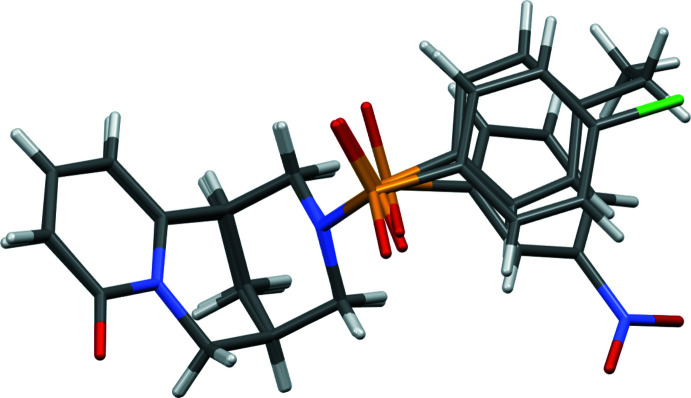
Overlay plot of the mol­ecules in the crystal structures of (**I**)–(**III**).

The asymmetric unit of (**I**) consists of one mol­ecule of (7*R*,9*R*)-*N*-[(4-ethyl­phen­yl)sulfon­yl]cytisine (Fig. 1 ▸). The methyl fragment (C8′*A*, C8′*B*) of the ethyl group bound to the phenyl ring is disordered over two sets of sites. In the crystal structures of (**II**) and (**III**), both asymmetric units likewise comprise one mol­ecule of (7*R*,9*R*)-*N*-[(4-chloro­phen­yl)sulfon­yl]cytisine and (7*R*,9*R*)-*N*-[(3-nitro­phen­yl)sulfon­yl]cytisine, respectively (Figs. 2 ▸, 3 ▸). The cytisine moieties in (**I**)–(**III**) are almost superimposable in the three mol­ecules (Fig. 4 ▸). Basically, the difference in the mol­ecular structures pertains to the arrangement of two fragments around the sulfonyl site, *i.e.* the arrangement of fragments along the S1—N12 and S1—C1′ bonds). Corresponding torsion angles C1′—S1—N12—C11 and N12—S1—C1′—C2′ are listed in Tables 1 ▸, 2 ▸ and 3 ▸.

## 3. Supra­molecular features

**Figure 5:**
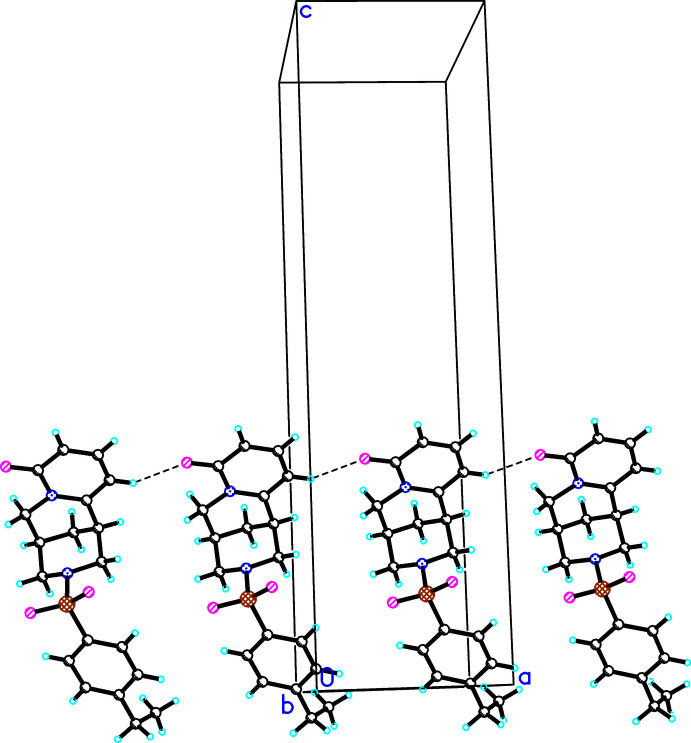
The observed C5—H⋯O1 hydrogen bond in the crystal structure of (**I**). For clarity, the disordered methyl fragment is not shown.

**Figure 7:**
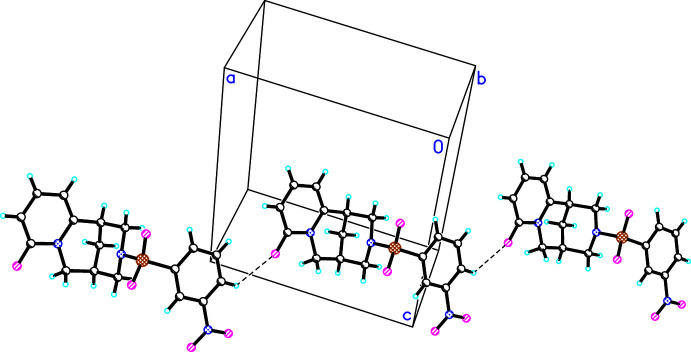
The observed hydrogen bonds (C4′—H⋯O1) in the crystal structure of (**III**).

In the crystal packing of (**I**)–(**III**), weak inter­molecular hydrogen bonds of the type C—H⋯O(C) are developed. In the crystal structures of (**I**) and (**II**), C—H⋯O1 hydrogen bonds link mol­ecules into chains directed parallel to [100] (Figs. 5 ▸, 6 ▸), besides other C—H⋯O or C—H⋯Cl (in the case of **II**) inter­actions (Tables 4 ▸, 5 ▸). In the crystal structure of (**III**), the C—H⋯O1 inter­actions link the mol­ecules into a chain running along [1 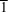 0] (Fig. 7 ▸, Table 6 ▸).

In order to visualize and qu­antify inter­molecular inter­actions in (**I**)–(**III**), a Hirshfeld surface analysis (Spackman & Jayatilaka, 2009 ▸) was performed with *Crystal Explorer 21* (Spackman *et al.*, 2021 ▸), and the associated two-dimensional fingerprint plots (McKinnon *et al.*, 2007 ▸) generated.

**Figure 8:**
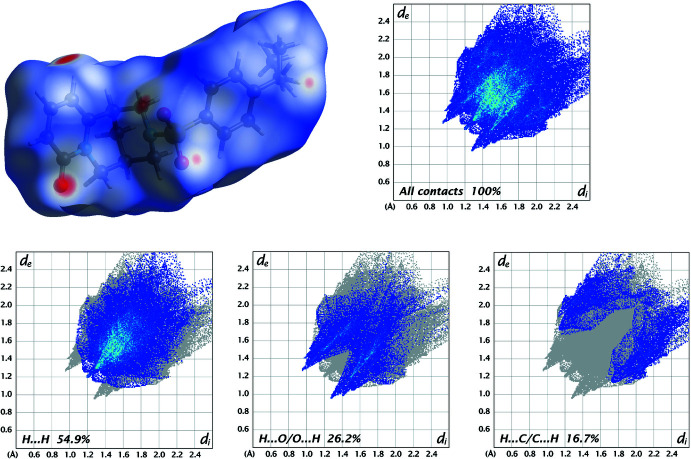
Three-dimensional Hirshfeld surfaces of the compound (**I**) plotted over *d*_norm_ in the range −0.2931 to 1.5624 a.u., and Hirshfeld fingerprint plots for all contacts and decomposed into H⋯H, H⋯O/O⋯H and H⋯C/C⋯H contacts. *d*_i_ and *d*_e_ denote the closest inter­nal and external distances (in Å) from a point on the surface.

The Hirshfeld surfaces for the mol­ecules in (**I**)–(**III**) are shown in Figs. 8 ▸–10 ▸ ▸ in which the two-dimensional fingerprint plots of the most dominant contacts are also presented.

For structure (**I**), H⋯H contacts are responsible for the largest contribution (54.9%) to the Hirshfeld surface. Besides these contacts, H⋯O/O⋯H (26.2%) and H⋯C/C⋯H (16.7%) inter­actions contribute significantly to the total Hirshfeld surface (Fig. 8 ▸). The contributions of further contacts are only minor and amount to H⋯N/N⋯H (1.8%), C⋯C (0.2%) and H⋯S/S⋯H (0.1%).

**Figure 9:**
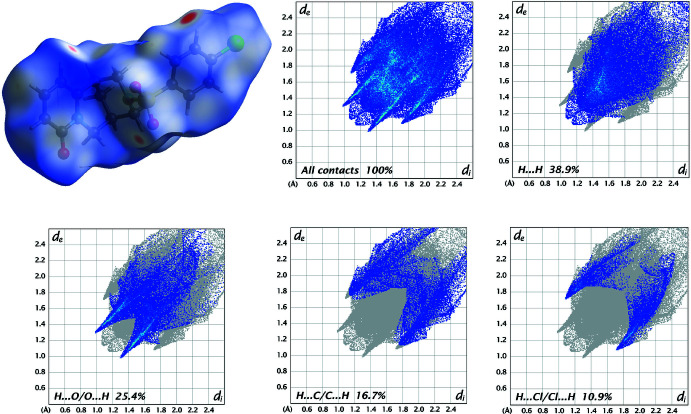
Three-dimensional Hirshfeld surfaces of the compound (**II**) plotted over *d*_norm_ in the range −0.2332 to 1.6350 a.u., and Hirshfeld fingerprint plots for all contacts and decomposed into H⋯H, H⋯O/O⋯H, H⋯C/C⋯H and H⋯Cl/Cl⋯H contacts. *d*_i_ and *d*_e_ denote the closest inter­nal and external distances (in Å) from a point on the surface.

In structure (**II**), the contribution percentages of the most significant contacts change because of the presence of H⋯Cl/Cl⋯H inter­actions and amount to H⋯H (38.9%), H⋯O/O⋯H (25.4%), H⋯C/C⋯H (16.7%) and H⋯Cl/Cl⋯H (10.9%) (Fig. 9 ▸). The contributions of further contacts are only minor and are Cl⋯O/O⋯Cl (2.4%), Cl⋯C/C⋯Cl (1.8%), C⋯O/O⋯C (1.7%), H⋯N/N⋯H (1.6%), C⋯C (0.3%) and Cl⋯S/S⋯Cl (0.1%).

**Figure 10:**
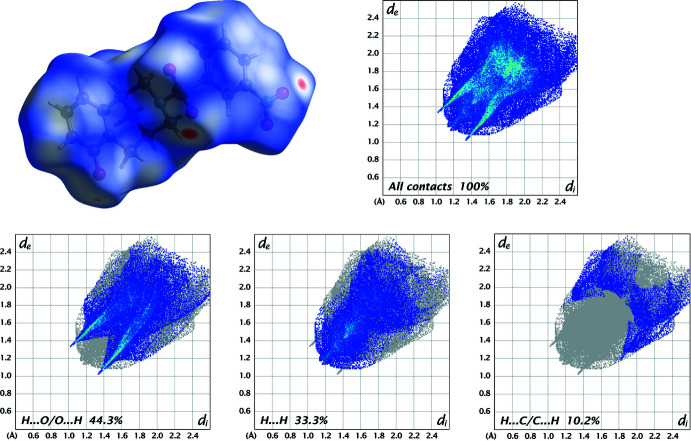
Three-dimensional Hirshfeld surfaces of the compound (**III**) plotted over *d*_norm_ in the range −0.1815 to 1.3331 a.u., and Hirshfeld fingerprint plots for all contacts and decomposed into H⋯O/O⋯H, H⋯H and H⋯C/C⋯H contacts. *d*_i_ and *d*_e_ denote the closest inter­nal and external distances (in Å) from a point on the surface.

In structure (**III**), the existence of a nitro group likewise changes the contributions of the significant inter­actions: H⋯O/O⋯H (44.3%), H⋯H (33.3%) and H⋯C/C⋯H (10.2%) (Fig. 10 ▸). Other minor contributions amount to C⋯C (3.8%), C⋯O/O⋯C (3.2%), H⋯N/N⋯H (2.5%), O⋯N/N⋯O (1.3%), O⋯O (1.2%) and C⋯N/N⋯C (0.2%).

## 4. Database survey

A Cambridge Structural Database search (version 2022.3.0; Groom *et al.*, 2016 ▸) revealed 99 *N*-derivatives of cytisine, of which twelve are *N*-benzyl derivatives of cytisine. *N*-aryl­sulfonyl­cytisine derivatives are not found. The most similar structure with respect to (**I**)–(**III**) is 3-[(4-bromo­phen­yl)meth­yl]-8-oxo-1,3,4,5,6,8-hexa­hydro-2*H*-1,5-methano­pyrido[1,2-*a*][1,5]diazo­cin-3-ium perchlorate (KINBOB; Przybył *et al.*, 2019 ▸).

## 5. Synthesis and crystallization

**General method**

**Figure 11:**
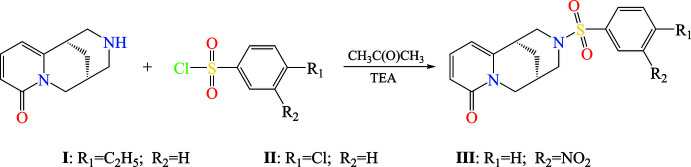
General reaction scheme for the synthesis of *N*-aryl­sulfonyl derivatives of cytisine.

Aryl­sulfonyl chloride (0.01 mol) and 10 ml of acetone were placed in a two-necked flask with a volume of 50 ml. After cooling, a previously prepared solution (1.9 g (0.01 mol) of cytisine and 0.01 mol of tri­ethyl­amine in 15 ml of acetone) was added under stirring through a separatory funnel. The reaction mixture was stirred at room temperature for 10 h. The reaction mixture was then left in the open air overnight to produce a dry mass. The mass was treated with 15 ml of distilled water and the remaining solid filtered off and dried in air. The reaction scheme is shown in Fig. 11 ▸.

**(7** ***R*** **,9** ***R*** **)-** ***N*** **-[(4-ethyl­phen­yl)sulfon­yl]cytisine (I)**

Yield 64% (2.29 g), m.p. 456–458 K, *R*_f_ = 0.59 [5:1 (*v*/*v*) benzene–ethanol].

**(7** ***R*** **,9** ***R*** **)-** ***N*** **-[(4-chloro­phen­yl)sulfon­yl]cytisine (II)**

Yield 76% (2.77 g), m.p. 488–490 K, *R*_f_ = 0.71 [5:1 (*v*/*v*) benzene–ethanol].

**(7** ***R*** **,9** ***R*** **)-** ***N*** **-[(3-nitro­phen­yl)sulfon­yl]cytisine (III)**

Yield 72% (2.71 g), m.p. 524–526 K, *R*_f_ = 0.50 [5:1 (*v*/*v*) benzene–ethanol].

Colourless crystals of (**I**)–(**III**) suitable for X-ray analysis were obtained by slow evaporation of an ethanol solution.

## 6. Refinement

Crystal data, data collection and structure refinement details are summarized in Table 7 ▸. In (**I**), the methyl C8′ atom is disordered over two positions (C8′*A*, C8′*B*). The site occupancy factors of the disordered fragment were refined with a free variable to a ratio of 0.55 (2):0.45 (2). Hydrogen atoms bonded to C atoms were placed geometrically (with C—H distances of 0.98 Å for CH, 0.97 Å for CH_2_, 0.96 Å for CH_3_ and 0.93 Å for C_ar_) and included in the refinement in a riding motion approximation with *U*_iso_(H) = 1.2U_eq_(C) or *U*_iso_ = 1.5*U*_eq_(C) for methyl H atoms.

## Supplementary Material

Crystal structure: contains datablock(s) I, II, III, Global. DOI: 10.1107/S2056989023001950/wm5674sup1.cif

Structure factors: contains datablock(s) I. DOI: 10.1107/S2056989023001950/wm5674Isup2.hkl

Structure factors: contains datablock(s) II. DOI: 10.1107/S2056989023001950/wm5674IIsup3.hkl

Structure factors: contains datablock(s) III. DOI: 10.1107/S2056989023001950/wm5674IIIsup4.hkl

CCDC references: 2245793, 2245792, 2245791

Additional supporting information:  crystallographic information; 3D view; checkCIF report

## Figures and Tables

**Figure 3:**
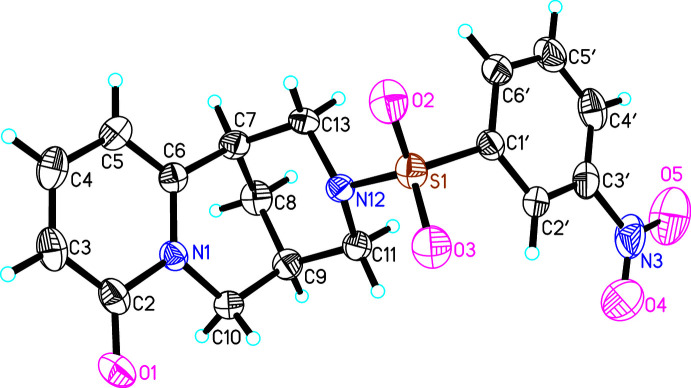
The asymmetric unit of (**III**) with atom labelling. Displacement ellipsoids represent 30% probability levels.

**Figure 6:**
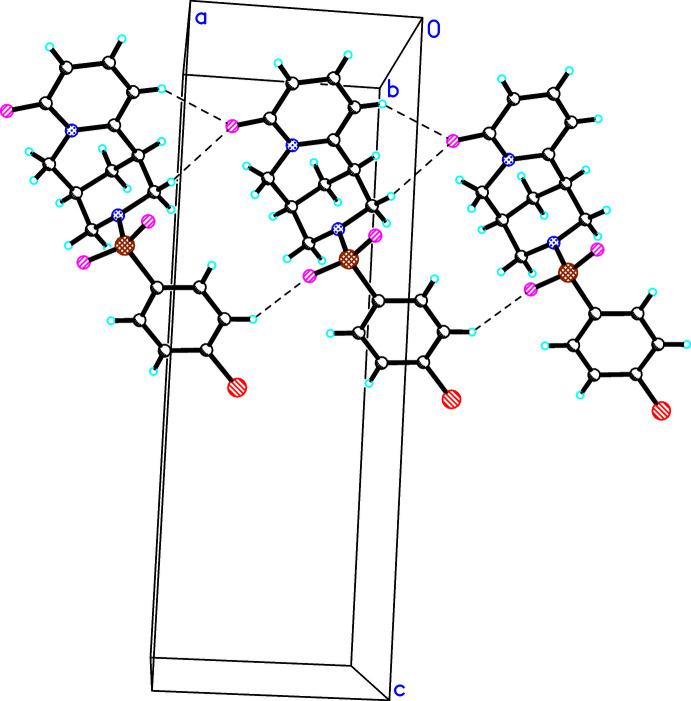
The observed hydrogen bonds (C5—H⋯O1, C13—H⋯O1, C5′—H⋯O3) in the crystal structure of (**II**).

**Table 1:** Selected torsion angles (°) for (**I**)

C1′—S1—N12—C11	76.6 (2)	N12—S1—C1′—C2′	−79.4 (2)

**Table 2:** Selected torsion angles (°) for (**II**)

C1′—S1—N12—C11	72.1 (2)	N12—S1—C1′—C2′	−79.4 (2)

**Table 3:** Selected torsion angles (°) for (**III**)

C1′—S1—N12—C11	57.3 (3)	N12—S1—C1′—C2′	−87.9 (3)

**Table 4:** Hydrogen-bond geometry (Å, °) for (**I**)

*D*—H⋯*A*	*D*—H	H⋯*A*	*D*⋯*A*	*D*—H⋯*A*
C5—H5*A*⋯O1^i^	0.93	2.34	3.116 (3)	141
C7—H7*A*⋯O2^ii^	0.98	2.44	3.254 (3)	140

Symmetry codes: (i) 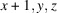 ; (ii) 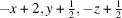 .

**Table 5:** Hydrogen-bond geometry (Å, °) for (**II**)

*D*—H⋯*A*	*D*—H	H⋯*A*	*D*⋯*A*	*D*—H⋯*A*
C5—H5*A*⋯O1^i^	0.93	2.61	3.375 (4)	140
C13—H13*B*⋯O1^i^	0.97	2.69	3.475 (3)	139
C5′—H5′*A*⋯O3^i^	0.93	2.42	3.198 (3)	142
C8—H8*A*⋯O3^ii^	0.97	2.56	3.424 (3)	149
C4—H4*A*⋯Cl1^iii^	0.93	2.94	3.764 (3)	148

Symmetry codes: (i) 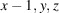 ; (ii) 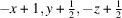 ; (iii) 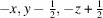 .

**Table 6:** Hydrogen-bond geometry (Å, °) for (**III**)

*D*—H⋯*A*	*D*—H	H⋯*A*	*D*⋯*A*	*D*—H⋯*A*
C4′—H4′*A*⋯O1^i^	0.93	2.61	3.257 (5)	127
C10—H10*A*⋯O5^ii^	0.97	2.58	3.460 (6)	151
C11—H11*A*⋯O5^iii^	0.97	2.55	3.428 (7)	150
C13—H13*A*⋯O3^iv^	0.97	2.46	3.330 (4)	150

Symmetry codes: (i) 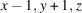 ; (ii) 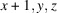 ; (iii) 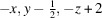 ; (iv) 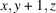 .

**Table 7:** Experimental details

	(**I**)	(**II**)	(**III**)
Crystal data			
Chemical formula	C_19_H_22_N_2_O_3_S	C_17_H_17_ClN_2_O_3_S	C_17_H_17_N_3_O_5_S
*M*_r_	358.44	364.83	375.39
Crystal system, space group	Orthorhombic, *P*2_1_2_1_2_1_	Orthorhombic, *P*2_1_2_1_2_1_	Monoclinic, *P*2_1_
Temperature (K)	299	299	296
*a*, *b*, *c* (Å)	6.9503 (14), 10.585 (2), 24.975 (5)	7.1374 (14), 11.448 (2), 20.844 (4)	11.040 (2), 6.2621 (13), 12.424 (3)
α, β, γ (°)	90, 90, 90	90, 90, 90	90, 94.03 (3), 90
*V* (Å^3^)	1837.5 (6)	1703.2 (6)	856.8 (3)
*Z*	4	4	2
Radiation type	Cu *K*α	Cu *K*α	Cu *K*α
μ (mm^−1^)	1.73	3.29	2.00
Crystal size (mm)	0.25 × 0.20 × 0.10	0.30 × 0.20 × 0.15	0.20 × 0.15 × 0.10

Data collection			
Diffractometer	XtaLAB Synergy, Single source at home/near, HyPix3000	XtaLAB Synergy, Single source at home/near, HyPix3000	XtaLAB Synergy, Single source at home/near, HyPix3000
Absorption correction	Multi-scan (*SADABS*; Krause *et al.*, 2015 ▸)	Multi-scan (*SADABS*; Krause *et al.*, 2015 ▸)	Multi-scan (*SADABS*; Krause *et al.*, 2015 ▸)
*T*_min_, *T*_max_	0.032, 1.000	0.795, 1.000	0.639, 1.000
No. of measured, independent and observed [*I* > 2σ(*I*)] reflections	17786, 3553, 3348	16028, 3289, 3203	7776, 2446, 2363
*R*_int_	0.054	0.027	0.023
(sin θ/λ)_max_ (Å^−1^)	0.615	0.615	0.615

Refinement			
*R*[*F* ^2^ > 2σ(*F* ^2^)], *wR*(*F* ^2^), *S*	0.038, 0.094, 1.11	0.030, 0.078, 1.05	0.039, 0.114, 1.04
No. of reflections	3553	3289	2446
No. of parameters	238	217	235
No. of restraints	0	0	1
H-atom treatment	H-atom parameters constrained	H-atom parameters constrained	H-atom parameters constrained
Δρ_max_, Δρ_min_ (e Å^−3^)	0.22, −0.44	0.42, −0.33	0.47, −0.22
Absolute structure	Flack *x* determined using 1319 quotients [(*I* ^+^)−(*I* ^−^)]/[(*I* ^+^)+(*I* ^−^)] (Parsons *et al.*, 2013 ▸)	Flack *x* determined using 1318 quotients [(*I* ^+^)−(*I* ^−^)]/[(*I* ^+^)+(*I* ^−^)] (Parsons *et al.*, 2013 ▸)	Flack *x* determined using 579 quotients [(*I* ^+^)−(*I* ^−^)]/[(*I* ^+^)+(*I* ^−^)] (Parsons *et al.*, 2013 ▸)
Absolute structure parameter	0.012 (9)	−0.004 (5)	0.017 (16)

Computer programs: *CrysAlis PRO* (Rigaku OD, 2021 ▸), *SHELXS7* (Sheldrick, 2008 ▸), *SHELXL2018/3* (Sheldrick, 2015 ▸), *XP* in *SHELXTL* (Sheldrick, 2008 ▸), *Mercury* (Macrae *et al.*, 2020 ▸), *PLATON* (Spek, 2020 ▸) and *publCIF* (Westrip, 2010 ▸).

## supplementary crystallographic information

### (1R,5R)-3-[(4-Ethylphenyl)sulfonyl]-1,2,3,4,5,6-hexahydro-8H-1,5-methanopyrido[1,2-a][1,5]diazocin-8-one (I) Crystal data

**Table:**

C_19_H_22_N_2_O_3_S	*D*_x_ = 1.296 Mg m^−^^3^
*M**_r_* = 358.44	Cu *K*α radiation, λ = 1.54184 Å
Orthorhombic, *P*2_1_2_1_2_1_	Cell parameters from 10591 reflections
*a* = 6.9503 (14) Å	θ = 3.5–70.9°
*b* = 10.585 (2) Å	µ = 1.73 mm^−^^1^
*c* = 24.975 (5) Å	*T* = 299 K
*V* = 1837.5 (6) Å^3^	Prizmatic, colorless
*Z* = 4	0.25 × 0.20 × 0.10 mm
*F*(000) = 760	

### (1R,5R)-3-[(4-Ethylphenyl)sulfonyl]-1,2,3,4,5,6-hexahydro-8H-1,5-methanopyrido[1,2-a][1,5]diazocin-8-one (I) Data collection

**Table:**

XtaLAB Synergy, Single source at home/near, HyPix3000 diffractometer	3553 independent reflections
Radiation source: micro-focus sealed X-ray tube, PhotonJet (Cu) X-ray Source	3348 reflections with *I* > 2σ(*I*)
Detector resolution: 10.0000 pixels mm^-1^	*R*_int_ = 0.054
ω scans	θ_max_ = 71.4°, θ_min_ = 3.5°
Absorption correction: multi-scan (SADABS; Krause *et al.*, 2015)	*h* = −8→8
*T*_min_ = 0.032, *T*_max_ = 1.000	*k* = −12→13
17786 measured reflections	*l* = −23→30

### (1R,5R)-3-[(4-Ethylphenyl)sulfonyl]-1,2,3,4,5,6-hexahydro-8H-1,5-methanopyrido[1,2-a][1,5]diazocin-8-one (I) Refinement

**Table:**

Refinement on *F*^2^	Hydrogen site location: inferred from neighbouring sites
Least-squares matrix: full	H-atom parameters constrained
*R*[*F*^2^ > 2σ(*F*^2^)] = 0.038	*w* = 1/[σ^2^(*F*_o_^2^) + (0.0523*P*)^2^ + 0.0534*P*] where *P* = (*F*_o_^2^ + 2*F*_c_^2^)/3
*wR*(*F*^2^) = 0.094	(Δ/σ)_max_ < 0.001
*S* = 1.11	Δρ_max_ = 0.22 e Å^−^^3^
3553 reflections	Δρ_min_ = −0.44 e Å^−^^3^
238 parameters	Absolute structure: Flack *x* determined using 1319 quotients [(*I*^+^)-(*I*^-^)]/[(*I*^+^)+(*I*^-^)] (Parsons *et al.*, 2013)
0 restraints	Absolute structure parameter: 0.012 (9)

### (1R,5R)-3-[(4-Ethylphenyl)sulfonyl]-1,2,3,4,5,6-hexahydro-8H-1,5-methanopyrido[1,2-a][1,5]diazocin-8-one (I) Special details

**Table:**

Geometry. All esds (except the esd in the dihedral angle between two l.s. planes) are estimated using the full covariance matrix. The cell esds are taken into account individually in the estimation of esds in distances, angles and torsion angles; correlations between esds in cell parameters are only used when they are defined by crystal symmetry. An approximate (isotropic) treatment of cell esds is used for estimating esds involving l.s. planes.

### (1R,5R)-3-[(4-Ethylphenyl)sulfonyl]-1,2,3,4,5,6-hexahydro-8H-1,5-methanopyrido[1,2-a][1,5]diazocin-8-one (I) Fractional atomic coordinates and isotropic or equivalent isotropic displacement parameters (Å^2^)

**Table:**

	*x*	*y*	*z*	*U*_iso_*/*U*_eq_	Occ. (<1)
S1	0.72079 (9)	0.74770 (5)	0.14537 (2)	0.05691 (18)	
O1	0.3969 (2)	0.73366 (18)	0.35879 (6)	0.0609 (4)	
O2	0.8449 (3)	0.64623 (16)	0.16065 (7)	0.0707 (5)	
O3	0.5240 (3)	0.7222 (2)	0.13301 (8)	0.0766 (6)	
N1	0.6396 (3)	0.83845 (17)	0.31683 (7)	0.0442 (4)	
N12	0.7204 (3)	0.84820 (18)	0.19511 (7)	0.0525 (4)	
C2	0.5717 (3)	0.7474 (2)	0.35284 (8)	0.0489 (5)	
C3	0.7162 (4)	0.6760 (3)	0.38004 (10)	0.0620 (6)	
H3A	0.679416	0.612285	0.403557	0.074*	
C4	0.9054 (4)	0.6993 (3)	0.37223 (11)	0.0660 (7)	
H4A	0.996719	0.652682	0.390946	0.079*	
C5	0.9646 (4)	0.7917 (3)	0.33673 (10)	0.0605 (6)	
H5A	1.095137	0.807418	0.331995	0.073*	
C6	0.8326 (3)	0.8596 (2)	0.30875 (9)	0.0492 (5)	
C7	0.8915 (4)	0.9562 (3)	0.26785 (10)	0.0585 (6)	
H7A	1.017190	0.990569	0.278075	0.070*	
C8	0.7474 (5)	1.0639 (2)	0.26561 (11)	0.0692 (7)	
H8A	0.736511	1.104049	0.300400	0.083*	
H8B	0.788228	1.126854	0.239766	0.083*	
C9	0.5559 (4)	1.0075 (3)	0.24915 (11)	0.0612 (7)	
H9A	0.461650	1.076197	0.247158	0.073*	
C10	0.4861 (3)	0.9134 (2)	0.29083 (10)	0.0524 (5)	
H10A	0.416201	0.959033	0.318316	0.063*	
H10B	0.396456	0.855705	0.273849	0.063*	
C11	0.5739 (4)	0.9493 (3)	0.19342 (10)	0.0641 (7)	
H11A	0.451036	0.914628	0.182325	0.077*	
H11B	0.611563	1.013563	0.167793	0.077*	
C13	0.9099 (4)	0.8943 (3)	0.21250 (10)	0.0592 (6)	
H13A	0.958528	0.955410	0.186892	0.071*	
H13B	1.000046	0.824482	0.214244	0.071*	
C1'	0.8285 (4)	0.8251 (2)	0.09016 (9)	0.0585 (6)	
C2'	0.7190 (5)	0.9095 (3)	0.06041 (11)	0.0780 (8)	
H2'A	0.590106	0.923114	0.068566	0.094*	
C3'	0.8057 (7)	0.9728 (3)	0.01842 (12)	0.0894 (11)	
H3'A	0.732521	1.029034	−0.001706	0.107*	
C4'	0.9946 (6)	0.9558 (3)	0.00544 (11)	0.0819 (10)	
C5'	1.1005 (6)	0.8709 (3)	0.03537 (12)	0.0849 (9)	
H5'A	1.229082	0.857186	0.026912	0.102*	
C6'	1.0180 (5)	0.8052 (3)	0.07808 (11)	0.0712 (7)	
H6'A	1.091003	0.748680	0.098065	0.085*	
C7'	1.0838 (8)	1.0285 (4)	−0.04059 (14)	0.1161 (17)	
H7'A	1.164041	0.971279	−0.061123	0.139*	0.55 (2)
H7'B	0.981596	1.057716	−0.063967	0.139*	0.55 (2)
H7'C	1.053155	0.983559	−0.073354	0.139*	0.45 (2)
H7'D	1.019922	1.109830	−0.042531	0.139*	0.45 (2)
C8'A	1.199 (3)	1.1360 (16)	−0.0246 (4)	0.136 (7)	0.55 (2)
H8'A	1.233872	1.184056	−0.055681	0.205*	0.55 (2)
H8'B	1.312957	1.107082	−0.006780	0.205*	0.55 (2)
H8'C	1.125652	1.188309	−0.000638	0.205*	0.55 (2)
C8'B	1.280 (2)	1.050 (2)	−0.0404 (4)	0.111 (5)	0.45 (2)
H8'D	1.312947	1.105567	−0.069535	0.166*	0.45 (2)
H8'E	1.347397	0.971814	−0.044349	0.166*	0.45 (2)
H8'F	1.316353	1.089349	−0.007150	0.166*	0.45 (2)

### (1R,5R)-3-[(4-Ethylphenyl)sulfonyl]-1,2,3,4,5,6-hexahydro-8H-1,5-methanopyrido[1,2-a][1,5]diazocin-8-one (I) Atomic displacement parameters (Å^2^)

**Table:**

	*U*^11^	*U*^22^	*U*^33^	*U*^12^	*U*^13^	*U*^23^
S1	0.0714 (4)	0.0452 (3)	0.0542 (3)	−0.0046 (3)	0.0012 (2)	0.0040 (2)
O1	0.0488 (9)	0.0641 (10)	0.0699 (10)	−0.0067 (8)	0.0057 (7)	0.0023 (9)
O2	0.0982 (14)	0.0453 (9)	0.0687 (10)	0.0091 (9)	0.0036 (10)	0.0092 (8)
O3	0.0790 (13)	0.0775 (13)	0.0733 (11)	−0.0218 (11)	−0.0037 (9)	−0.0046 (10)
N1	0.0400 (9)	0.0428 (9)	0.0498 (9)	0.0003 (7)	−0.0009 (7)	−0.0053 (7)
N12	0.0558 (11)	0.0508 (10)	0.0510 (9)	0.0010 (9)	0.0009 (9)	0.0024 (8)
C2	0.0483 (11)	0.0486 (11)	0.0499 (10)	−0.0027 (10)	−0.0015 (8)	−0.0064 (11)
C3	0.0698 (16)	0.0617 (14)	0.0546 (12)	0.0022 (13)	−0.0104 (12)	0.0050 (11)
C4	0.0597 (15)	0.0767 (17)	0.0614 (13)	0.0145 (13)	−0.0193 (11)	−0.0041 (13)
C5	0.0406 (11)	0.0813 (18)	0.0595 (12)	0.0023 (11)	−0.0057 (10)	−0.0113 (12)
C6	0.0419 (11)	0.0543 (12)	0.0514 (11)	−0.0040 (10)	0.0013 (9)	−0.0133 (10)
C7	0.0522 (13)	0.0583 (14)	0.0649 (14)	−0.0145 (11)	0.0065 (11)	−0.0104 (11)
C8	0.088 (2)	0.0459 (12)	0.0741 (15)	−0.0106 (14)	0.0117 (15)	−0.0046 (11)
C9	0.0679 (17)	0.0469 (13)	0.0689 (15)	0.0121 (12)	0.0039 (14)	0.0033 (11)
C10	0.0443 (11)	0.0503 (12)	0.0625 (13)	0.0082 (10)	−0.0002 (10)	−0.0019 (10)
C11	0.0740 (17)	0.0561 (14)	0.0623 (14)	0.0117 (13)	−0.0004 (13)	0.0086 (12)
C13	0.0566 (13)	0.0607 (14)	0.0603 (13)	−0.0092 (11)	0.0106 (11)	0.0014 (11)
C1'	0.0768 (17)	0.0495 (12)	0.0492 (11)	−0.0015 (12)	0.0001 (11)	0.0000 (10)
C2'	0.087 (2)	0.0819 (19)	0.0648 (15)	0.0054 (18)	−0.0035 (15)	0.0168 (14)
C3'	0.126 (3)	0.079 (2)	0.0635 (17)	0.003 (2)	−0.0097 (19)	0.0196 (15)
C4'	0.125 (3)	0.0704 (19)	0.0503 (14)	−0.0161 (19)	0.0094 (17)	0.0020 (13)
C5'	0.094 (2)	0.093 (2)	0.0684 (17)	−0.0076 (19)	0.0210 (16)	−0.0031 (16)
C6'	0.0838 (19)	0.0683 (16)	0.0615 (14)	0.0077 (15)	0.0101 (14)	0.0040 (13)
C7'	0.182 (5)	0.106 (3)	0.0603 (18)	−0.035 (3)	0.018 (2)	0.0090 (19)
C8'A	0.219 (15)	0.128 (10)	0.062 (4)	−0.069 (11)	0.000 (6)	0.020 (5)
C8'B	0.130 (9)	0.134 (12)	0.069 (5)	−0.043 (8)	0.023 (5)	0.010 (7)

### (1R,5R)-3-[(4-Ethylphenyl)sulfonyl]-1,2,3,4,5,6-hexahydro-8H-1,5-methanopyrido[1,2-a][1,5]diazocin-8-one (I) Geometric parameters (Å, º)

**Table:**

S1—O3	1.428 (2)	C10—H10B	0.9700
S1—O2	1.429 (2)	C11—H11A	0.9700
S1—N12	1.635 (2)	C11—H11B	0.9700
S1—C1'	1.770 (3)	C13—H13A	0.9700
O1—C2	1.233 (3)	C13—H13B	0.9700
N1—C6	1.375 (3)	C1'—C6'	1.368 (4)
N1—C2	1.400 (3)	C1'—C2'	1.389 (4)
N1—C10	1.479 (3)	C2'—C3'	1.383 (5)
N12—C13	1.471 (3)	C2'—H2'A	0.9300
N12—C11	1.478 (3)	C3'—C4'	1.365 (6)
C2—C3	1.429 (3)	C3'—H3'A	0.9300
C3—C4	1.352 (4)	C4'—C5'	1.381 (5)
C3—H3A	0.9300	C4'—C7'	1.515 (4)
C4—C5	1.383 (4)	C5'—C6'	1.396 (4)
C4—H4A	0.9300	C5'—H5'A	0.9300
C5—C6	1.359 (3)	C6'—H6'A	0.9300
C5—H5A	0.9300	C7'—C8'B	1.385 (12)
C6—C7	1.502 (4)	C7'—C8'A	1.447 (11)
C7—C8	1.519 (4)	C7'—H7'A	0.9700
C7—C13	1.535 (3)	C7'—H7'B	0.9700
C7—H7A	0.9800	C7'—H7'C	0.9700
C8—C9	1.516 (4)	C7'—H7'D	0.9700
C8—H8A	0.9700	C8'A—H8'A	0.9600
C8—H8B	0.9700	C8'A—H8'B	0.9600
C9—C10	1.520 (4)	C8'A—H8'C	0.9600
C9—C11	1.527 (4)	C8'B—H8'D	0.9600
C9—H9A	0.9800	C8'B—H8'E	0.9600
C10—H10A	0.9700	C8'B—H8'F	0.9600
			
O3—S1—O2	119.57 (13)	C9—C11—H11A	109.9
O3—S1—N12	106.61 (11)	N12—C11—H11B	109.9
O2—S1—N12	106.68 (10)	C9—C11—H11B	109.9
O3—S1—C1'	108.91 (13)	H11A—C11—H11B	108.3
O2—S1—C1'	107.49 (13)	N12—C13—C7	109.45 (19)
N12—S1—C1'	106.94 (11)	N12—C13—H13A	109.8
C6—N1—C2	122.35 (18)	C7—C13—H13A	109.8
C6—N1—C10	123.50 (19)	N12—C13—H13B	109.8
C2—N1—C10	114.07 (17)	C7—C13—H13B	109.8
C13—N12—C11	112.7 (2)	H13A—C13—H13B	108.2
C13—N12—S1	116.04 (16)	C6'—C1'—C2'	120.6 (3)
C11—N12—S1	116.78 (16)	C6'—C1'—S1	120.5 (2)
O1—C2—N1	119.4 (2)	C2'—C1'—S1	118.9 (2)
O1—C2—C3	125.0 (2)	C3'—C2'—C1'	118.6 (3)
N1—C2—C3	115.6 (2)	C3'—C2'—H2'A	120.7
C4—C3—C2	121.2 (3)	C1'—C2'—H2'A	120.7
C4—C3—H3A	119.4	C4'—C3'—C2'	122.4 (3)
C2—C3—H3A	119.4	C4'—C3'—H3'A	118.8
C3—C4—C5	120.7 (2)	C2'—C3'—H3'A	118.8
C3—C4—H4A	119.6	C3'—C4'—C5'	118.0 (3)
C5—C4—H4A	119.6	C3'—C4'—C7'	120.4 (4)
C6—C5—C4	120.2 (2)	C5'—C4'—C7'	121.6 (4)
C6—C5—H5A	119.9	C4'—C5'—C6'	121.3 (3)
C4—C5—H5A	119.9	C4'—C5'—H5'A	119.4
C5—C6—N1	119.8 (2)	C6'—C5'—H5'A	119.4
C5—C6—C7	121.7 (2)	C1'—C6'—C5'	119.2 (3)
N1—C6—C7	118.5 (2)	C1'—C6'—H6'A	120.4
C6—C7—C8	110.9 (2)	C5'—C6'—H6'A	120.4
C6—C7—C13	110.2 (2)	C8'B—C7'—C4'	119.0 (6)
C8—C7—C13	110.0 (2)	C8'A—C7'—C4'	114.5 (5)
C6—C7—H7A	108.6	C8'A—C7'—H7'A	108.6
C8—C7—H7A	108.6	C4'—C7'—H7'A	108.6
C13—C7—H7A	108.6	C8'A—C7'—H7'B	108.6
C9—C8—C7	107.0 (2)	C4'—C7'—H7'B	108.6
C9—C8—H8A	110.3	H7'A—C7'—H7'B	107.6
C7—C8—H8A	110.3	C8'B—C7'—H7'C	107.6
C9—C8—H8B	110.3	C4'—C7'—H7'C	107.6
C7—C8—H8B	110.3	C8'B—C7'—H7'D	107.6
H8A—C8—H8B	108.6	C4'—C7'—H7'D	107.6
C8—C9—C10	110.6 (2)	H7'C—C7'—H7'D	107.0
C8—C9—C11	109.5 (2)	C7'—C8'A—H8'A	109.5
C10—C9—C11	112.7 (2)	C7'—C8'A—H8'B	109.5
C8—C9—H9A	107.9	H8'A—C8'A—H8'B	109.5
C10—C9—H9A	107.9	C7'—C8'A—H8'C	109.5
C11—C9—H9A	107.9	H8'A—C8'A—H8'C	109.5
N1—C10—C9	115.0 (2)	H8'B—C8'A—H8'C	109.5
N1—C10—H10A	108.5	C7'—C8'B—H8'D	109.5
C9—C10—H10A	108.5	C7'—C8'B—H8'E	109.5
N1—C10—H10B	108.5	H8'D—C8'B—H8'E	109.5
C9—C10—H10B	108.5	C7'—C8'B—H8'F	109.5
H10A—C10—H10B	107.5	H8'D—C8'B—H8'F	109.5
N12—C11—C9	108.8 (2)	H8'E—C8'B—H8'F	109.5
N12—C11—H11A	109.9		
			
O3—S1—N12—C13	−176.35 (18)	C8—C9—C10—N1	−35.6 (3)
O2—S1—N12—C13	54.8 (2)	C11—C9—C10—N1	87.4 (3)
C1'—S1—N12—C13	−60.0 (2)	C13—N12—C11—C9	−58.7 (3)
O3—S1—N12—C11	−39.7 (2)	S1—N12—C11—C9	163.28 (18)
O2—S1—N12—C11	−168.57 (19)	C8—C9—C11—N12	60.8 (3)
C1'—S1—N12—C11	76.6 (2)	C10—C9—C11—N12	−62.8 (3)
C6—N1—C2—O1	179.2 (2)	C11—N12—C13—C7	57.4 (3)
C10—N1—C2—O1	2.3 (3)	S1—N12—C13—C7	−164.26 (18)
C6—N1—C2—C3	−1.1 (3)	C6—C7—C13—N12	64.3 (3)
C10—N1—C2—C3	−178.03 (19)	C8—C7—C13—N12	−58.3 (3)
O1—C2—C3—C4	−178.3 (2)	O3—S1—C1'—C6'	−146.7 (2)
N1—C2—C3—C4	2.1 (3)	O2—S1—C1'—C6'	−15.8 (3)
C2—C3—C4—C5	−1.3 (4)	N12—S1—C1'—C6'	98.4 (2)
C3—C4—C5—C6	−0.7 (4)	O3—S1—C1'—C2'	35.4 (3)
C4—C5—C6—N1	1.7 (4)	O2—S1—C1'—C2'	166.3 (2)
C4—C5—C6—C7	−176.9 (2)	N12—S1—C1'—C2'	−79.4 (2)
C2—N1—C6—C5	−0.7 (3)	C6'—C1'—C2'—C3'	0.0 (5)
C10—N1—C6—C5	175.9 (2)	S1—C1'—C2'—C3'	177.9 (3)
C2—N1—C6—C7	177.89 (18)	C1'—C2'—C3'—C4'	−0.4 (5)
C10—N1—C6—C7	−5.5 (3)	C2'—C3'—C4'—C5'	0.7 (6)
C5—C6—C7—C8	−148.0 (2)	C2'—C3'—C4'—C7'	−179.3 (3)
N1—C6—C7—C8	33.4 (3)	C3'—C4'—C5'—C6'	−0.7 (5)
C5—C6—C7—C13	90.0 (3)	C7'—C4'—C5'—C6'	179.3 (3)
N1—C6—C7—C13	−88.6 (3)	C2'—C1'—C6'—C5'	0.0 (4)
C6—C7—C8—C9	−61.4 (3)	S1—C1'—C6'—C5'	−177.9 (2)
C13—C7—C8—C9	60.7 (3)	C4'—C5'—C6'—C1'	0.4 (5)
C7—C8—C9—C10	62.7 (3)	C3'—C4'—C7'—C8'B	154.4 (12)
C7—C8—C9—C11	−62.1 (3)	C5'—C4'—C7'—C8'B	−25.7 (12)
C6—N1—C10—C9	6.6 (3)	C3'—C4'—C7'—C8'A	101.1 (12)
C2—N1—C10—C9	−176.56 (19)	C5'—C4'—C7'—C8'A	−78.9 (12)

### (1R,5R)-3-[(4-Ethylphenyl)sulfonyl]-1,2,3,4,5,6-hexahydro-8H-1,5-methanopyrido[1,2-a][1,5]diazocin-8-one (I) Hydrogen-bond geometry (Å, º)

**Table:**

*D*—H···*A*	*D*—H	H···*A*	*D*···*A*	*D*—H···*A*
C5—H5*A*···O1^i^	0.93	2.34	3.116 (3)	141
C7—H7*A*···O2^ii^	0.98	2.44	3.254 (3)	140

Symmetry codes: (i) *x*+1, *y*, *z*; (ii) −*x*+2, *y*+1/2, −*z*+1/2.

### (1R,5R)-3-[(4-chlorophenyl)sulfonyl]-1,2,3,4,5,6-hexahydro-8H-1,5-methanopyrido[1,2-a][1,5]diazocin-8-one (II) Crystal data

**Table:**

C_17_H_17_ClN_2_O_3_S	*D*_x_ = 1.423 Mg m^−^^3^
*M**_r_* = 364.83	Cu *K*α radiation, λ = 1.54184 Å
Orthorhombic, *P*2_1_2_1_2_1_	Cell parameters from 12750 reflections
*a* = 7.1374 (14) Å	θ = 3.9–71.2°
*b* = 11.448 (2) Å	µ = 3.29 mm^−^^1^
*c* = 20.844 (4) Å	*T* = 299 K
*V* = 1703.2 (6) Å^3^	Prizmatic, colorless
*Z* = 4	0.30 × 0.20 × 0.15 mm
*F*(000) = 760	

### (1R,5R)-3-[(4-chlorophenyl)sulfonyl]-1,2,3,4,5,6-hexahydro-8H-1,5-methanopyrido[1,2-a][1,5]diazocin-8-one (II) Data collection

**Table:**

XtaLAB Synergy, Single source at home/near, HyPix3000 diffractometer	3289 independent reflections
Radiation source: micro-focus sealed X-ray tube, PhotonJet (Cu) X-ray Source	3203 reflections with *I* > 2σ(*I*)
Detector resolution: 10.0000 pixels mm^-1^	*R*_int_ = 0.027
ω scans	θ_max_ = 71.4°, θ_min_ = 4.2°
Absorption correction: multi-scan (SADABS; Krause *et al.*, 2015)	*h* = −8→8
*T*_min_ = 0.795, *T*_max_ = 1.000	*k* = −14→13
16028 measured reflections	*l* = −25→25

### (1R,5R)-3-[(4-chlorophenyl)sulfonyl]-1,2,3,4,5,6-hexahydro-8H-1,5-methanopyrido[1,2-a][1,5]diazocin-8-one (II) Refinement

**Table:**

Refinement on *F*^2^	Hydrogen site location: inferred from neighbouring sites
Least-squares matrix: full	H-atom parameters constrained
*R*[*F*^2^ > 2σ(*F*^2^)] = 0.030	*w* = 1/[σ^2^(*F*_o_^2^) + (0.0503*P*)^2^ + 0.1799*P*] where *P* = (*F*_o_^2^ + 2*F*_c_^2^)/3
*wR*(*F*^2^) = 0.078	(Δ/σ)_max_ < 0.001
*S* = 1.05	Δρ_max_ = 0.42 e Å^−^^3^
3289 reflections	Δρ_min_ = −0.33 e Å^−^^3^
217 parameters	Absolute structure: Flack *x* determined using 1318 quotients [(*I*^+^)-(*I*^-^)]/[(*I*^+^)+(*I*^-^)] (Parsons *et al.*, 2013)
0 restraints	Absolute structure parameter: −0.004 (5)

### (1R,5R)-3-[(4-chlorophenyl)sulfonyl]-1,2,3,4,5,6-hexahydro-8H-1,5-methanopyrido[1,2-a][1,5]diazocin-8-one (II) Special details

**Table:**

Geometry. All esds (except the esd in the dihedral angle between two l.s. planes) are estimated using the full covariance matrix. The cell esds are taken into account individually in the estimation of esds in distances, angles and torsion angles; correlations between esds in cell parameters are only used when they are defined by crystal symmetry. An approximate (isotropic) treatment of cell esds is used for estimating esds involving l.s. planes.

### (1R,5R)-3-[(4-chlorophenyl)sulfonyl]-1,2,3,4,5,6-hexahydro-8H-1,5-methanopyrido[1,2-a][1,5]diazocin-8-one (II) Fractional atomic coordinates and isotropic or equivalent isotropic displacement parameters (Å^2^)

**Table:**

	*x*	*y*	*z*	*U*_iso_*/*U*_eq_	
S1	0.22478 (8)	0.27207 (5)	0.34739 (3)	0.03902 (15)	
Cl1	−0.32153 (13)	0.49086 (7)	0.54785 (3)	0.0699 (2)	
O1	0.7771 (3)	0.28000 (19)	0.15317 (11)	0.0647 (5)	
O2	0.1269 (3)	0.18068 (15)	0.31475 (8)	0.0502 (4)	
O3	0.3999 (3)	0.24823 (16)	0.37824 (9)	0.0524 (4)	
N1	0.4944 (3)	0.36786 (17)	0.16841 (9)	0.0409 (4)	
N12	0.2648 (3)	0.37419 (16)	0.29491 (8)	0.0401 (4)	
C2	0.6102 (4)	0.2880 (2)	0.13668 (12)	0.0488 (6)	
C3	0.5244 (5)	0.2229 (3)	0.08706 (15)	0.0627 (7)	
H3A	0.596685	0.171265	0.063104	0.075*	
C4	0.3400 (5)	0.2340 (3)	0.07356 (14)	0.0655 (8)	
H4A	0.287153	0.189194	0.041014	0.079*	
C5	0.2283 (4)	0.3117 (3)	0.10791 (13)	0.0562 (6)	
H5A	0.100971	0.317377	0.098975	0.067*	
C6	0.3060 (3)	0.3793 (2)	0.15466 (11)	0.0423 (5)	
C7	0.1922 (3)	0.4659 (2)	0.19194 (12)	0.0451 (5)	
H7A	0.088762	0.492844	0.164794	0.054*	
C8	0.3121 (4)	0.5714 (2)	0.21021 (14)	0.0529 (6)	
H8A	0.363572	0.607885	0.172078	0.064*	
H8B	0.237218	0.628713	0.232979	0.064*	
C9	0.4687 (4)	0.5263 (2)	0.25296 (13)	0.0476 (5)	
H9A	0.546300	0.592999	0.265695	0.057*	
C10	0.5921 (3)	0.4407 (2)	0.21691 (13)	0.0471 (6)	
H10A	0.690567	0.484286	0.195534	0.057*	
H10B	0.651477	0.389329	0.247811	0.057*	
C11	0.3847 (4)	0.4731 (2)	0.31352 (12)	0.0480 (6)	
H11A	0.483789	0.446415	0.341787	0.058*	
H11B	0.311175	0.531235	0.336171	0.058*	
C13	0.1095 (3)	0.4089 (2)	0.25235 (12)	0.0451 (5)	
H13A	0.027946	0.463659	0.274269	0.054*	
H13B	0.036134	0.340816	0.240581	0.054*	
C1'	0.0721 (3)	0.3307 (2)	0.40561 (11)	0.0395 (5)	
C2'	0.1424 (4)	0.4029 (3)	0.45401 (13)	0.0536 (6)	
H2'A	0.270287	0.417502	0.456776	0.064*	
C3'	0.0214 (5)	0.4522 (3)	0.49743 (13)	0.0607 (7)	
H3'A	0.066574	0.501348	0.529405	0.073*	
C4'	−0.1678 (4)	0.4284 (2)	0.49336 (11)	0.0472 (6)	
C5'	−0.2378 (4)	0.3554 (3)	0.44671 (12)	0.0549 (6)	
H5'A	−0.365241	0.338706	0.445122	0.066*	
C6'	−0.1173 (4)	0.3073 (2)	0.40242 (12)	0.0496 (6)	
H6'A	−0.163668	0.259030	0.370264	0.060*	

### (1R,5R)-3-[(4-chlorophenyl)sulfonyl]-1,2,3,4,5,6-hexahydro-8H-1,5-methanopyrido[1,2-a][1,5]diazocin-8-one (II) Atomic displacement parameters (Å^2^)

**Table:**

	*U*^11^	*U*^22^	*U*^33^	*U*^12^	*U*^13^	*U*^23^
S1	0.0399 (3)	0.0367 (3)	0.0404 (3)	−0.0018 (2)	0.0007 (2)	−0.0021 (2)
Cl1	0.0922 (6)	0.0660 (4)	0.0514 (4)	0.0183 (4)	0.0234 (4)	−0.0014 (3)
O1	0.0432 (9)	0.0705 (12)	0.0805 (13)	0.0155 (9)	0.0077 (9)	0.0098 (11)
O2	0.0568 (10)	0.0399 (8)	0.0539 (9)	−0.0081 (7)	0.0038 (8)	−0.0110 (8)
O3	0.0469 (9)	0.0530 (10)	0.0572 (10)	0.0070 (8)	−0.0037 (8)	0.0050 (8)
N1	0.0363 (9)	0.0433 (10)	0.0432 (10)	0.0015 (8)	0.0017 (7)	0.0079 (8)
N12	0.0380 (9)	0.0428 (9)	0.0395 (9)	−0.0068 (8)	−0.0036 (8)	0.0006 (8)
C2	0.0471 (13)	0.0464 (13)	0.0530 (14)	0.0065 (10)	0.0087 (11)	0.0103 (10)
C3	0.0742 (19)	0.0554 (15)	0.0586 (15)	0.0143 (15)	0.0122 (14)	−0.0012 (14)
C4	0.077 (2)	0.0630 (17)	0.0562 (15)	0.0023 (16)	−0.0086 (14)	−0.0106 (14)
C5	0.0486 (14)	0.0666 (16)	0.0533 (14)	0.0015 (12)	−0.0082 (12)	−0.0014 (12)
C6	0.0393 (11)	0.0476 (12)	0.0398 (11)	0.0024 (9)	−0.0019 (9)	0.0099 (10)
C7	0.0398 (12)	0.0509 (13)	0.0446 (12)	0.0077 (10)	−0.0028 (9)	0.0080 (10)
C8	0.0580 (15)	0.0406 (12)	0.0602 (15)	0.0069 (11)	0.0042 (12)	0.0103 (11)
C9	0.0495 (13)	0.0387 (12)	0.0547 (13)	−0.0094 (10)	−0.0014 (12)	0.0027 (10)
C10	0.0369 (12)	0.0502 (13)	0.0543 (14)	−0.0047 (10)	−0.0028 (10)	0.0054 (11)
C11	0.0506 (13)	0.0461 (12)	0.0472 (12)	−0.0113 (11)	−0.0041 (11)	−0.0037 (10)
C13	0.0335 (10)	0.0559 (14)	0.0458 (12)	0.0017 (10)	−0.0003 (9)	0.0004 (10)
C1'	0.0424 (11)	0.0396 (11)	0.0365 (10)	−0.0049 (9)	0.0009 (9)	−0.0017 (9)
C2'	0.0494 (14)	0.0638 (15)	0.0477 (13)	−0.0126 (12)	−0.0036 (12)	−0.0117 (12)
C3'	0.0720 (18)	0.0653 (17)	0.0447 (14)	−0.0100 (15)	−0.0002 (13)	−0.0196 (13)
C4'	0.0609 (15)	0.0447 (12)	0.0360 (11)	0.0056 (11)	0.0060 (10)	0.0023 (10)
C5'	0.0441 (13)	0.0681 (16)	0.0527 (13)	−0.0021 (12)	0.0034 (11)	−0.0091 (12)
C6'	0.0454 (13)	0.0590 (15)	0.0444 (12)	−0.0114 (11)	−0.0008 (11)	−0.0125 (11)

### (1R,5R)-3-[(4-chlorophenyl)sulfonyl]-1,2,3,4,5,6-hexahydro-8H-1,5-methanopyrido[1,2-a][1,5]diazocin-8-one (II) Geometric parameters (Å, º)

**Table:**

S1—O2	1.4304 (17)	C8—C9	1.520 (4)
S1—O3	1.4319 (18)	C8—H8A	0.9700
S1—N12	1.6262 (19)	C8—H8B	0.9700
S1—C1'	1.764 (2)	C9—C10	1.517 (4)
Cl1—C4'	1.733 (3)	C9—C11	1.524 (4)
O1—C2	1.243 (3)	C9—H9A	0.9800
N1—C6	1.381 (3)	C10—H10A	0.9700
N1—C2	1.399 (3)	C10—H10B	0.9700
N1—C10	1.485 (3)	C11—H11A	0.9700
N12—C11	1.471 (3)	C11—H11B	0.9700
N12—C13	1.474 (3)	C13—H13A	0.9700
C2—C3	1.414 (4)	C13—H13B	0.9700
C3—C4	1.352 (5)	C1'—C6'	1.379 (3)
C3—H3A	0.9300	C1'—C2'	1.398 (3)
C4—C5	1.393 (4)	C2'—C3'	1.372 (4)
C4—H4A	0.9300	C2'—H2'A	0.9300
C5—C6	1.363 (4)	C3'—C4'	1.380 (4)
C5—H5A	0.9300	C3'—H3'A	0.9300
C6—C7	1.499 (3)	C4'—C5'	1.376 (4)
C7—C8	1.528 (4)	C5'—C6'	1.377 (4)
C7—C13	1.537 (3)	C5'—H5'A	0.9300
C7—H7A	0.9800	C6'—H6'A	0.9300
			
O2—S1—O3	120.05 (11)	C8—C9—C11	109.4 (2)
O2—S1—N12	106.96 (10)	C10—C9—H9A	108.0
O3—S1—N12	106.62 (11)	C8—C9—H9A	108.0
O2—S1—C1'	107.67 (11)	C11—C9—H9A	108.0
O3—S1—C1'	107.63 (11)	N1—C10—C9	115.3 (2)
N12—S1—C1'	107.33 (11)	N1—C10—H10A	108.4
C6—N1—C2	122.6 (2)	C9—C10—H10A	108.4
C6—N1—C10	123.0 (2)	N1—C10—H10B	108.4
C2—N1—C10	114.32 (19)	C9—C10—H10B	108.4
C11—N12—C13	112.9 (2)	H10A—C10—H10B	107.5
C11—N12—S1	118.55 (15)	N12—C11—C9	108.5 (2)
C13—N12—S1	117.80 (16)	N12—C11—H11A	110.0
O1—C2—N1	118.9 (3)	C9—C11—H11A	110.0
O1—C2—C3	125.4 (3)	N12—C11—H11B	110.0
N1—C2—C3	115.7 (2)	C9—C11—H11B	110.0
C4—C3—C2	121.6 (3)	H11A—C11—H11B	108.4
C4—C3—H3A	119.2	N12—C13—C7	108.57 (19)
C2—C3—H3A	119.2	N12—C13—H13A	110.0
C3—C4—C5	120.7 (3)	C7—C13—H13A	110.0
C3—C4—H4A	119.7	N12—C13—H13B	110.0
C5—C4—H4A	119.7	C7—C13—H13B	110.0
C6—C5—C4	119.9 (3)	H13A—C13—H13B	108.4
C6—C5—H5A	120.1	C6'—C1'—C2'	120.1 (2)
C4—C5—H5A	120.1	C6'—C1'—S1	119.92 (18)
C5—C6—N1	119.4 (2)	C2'—C1'—S1	120.00 (19)
C5—C6—C7	121.7 (2)	C3'—C2'—C1'	119.6 (2)
N1—C6—C7	118.9 (2)	C3'—C2'—H2'A	120.2
C6—C7—C8	110.4 (2)	C1'—C2'—H2'A	120.2
C6—C7—C13	110.61 (19)	C2'—C3'—C4'	119.6 (2)
C8—C7—C13	110.3 (2)	C2'—C3'—H3'A	120.2
C6—C7—H7A	108.5	C4'—C3'—H3'A	120.2
C8—C7—H7A	108.5	C5'—C4'—C3'	121.2 (2)
C13—C7—H7A	108.5	C5'—C4'—Cl1	118.9 (2)
C9—C8—C7	106.80 (19)	C3'—C4'—Cl1	119.8 (2)
C9—C8—H8A	110.4	C4'—C5'—C6'	119.3 (2)
C7—C8—H8A	110.4	C4'—C5'—H5'A	120.3
C9—C8—H8B	110.4	C6'—C5'—H5'A	120.3
C7—C8—H8B	110.4	C5'—C6'—C1'	120.2 (2)
H8A—C8—H8B	108.6	C5'—C6'—H6'A	119.9
C10—C9—C8	110.9 (2)	C1'—C6'—H6'A	119.9
C10—C9—C11	112.4 (2)		
			
O2—S1—N12—C11	−172.55 (18)	C6—N1—C10—C9	2.5 (3)
O3—S1—N12—C11	−43.0 (2)	C2—N1—C10—C9	−178.8 (2)
C1'—S1—N12—C11	72.1 (2)	C8—C9—C10—N1	−33.3 (3)
O2—S1—N12—C13	45.5 (2)	C11—C9—C10—N1	89.4 (3)
O3—S1—N12—C13	175.12 (17)	C13—N12—C11—C9	−60.2 (3)
C1'—S1—N12—C13	−69.78 (19)	S1—N12—C11—C9	156.07 (18)
C6—N1—C2—O1	−177.7 (2)	C10—C9—C11—N12	−62.0 (3)
C10—N1—C2—O1	3.6 (3)	C8—C9—C11—N12	61.6 (3)
C6—N1—C2—C3	3.0 (3)	C11—N12—C13—C7	58.3 (3)
C10—N1—C2—C3	−175.7 (2)	S1—N12—C13—C7	−157.68 (17)
O1—C2—C3—C4	177.7 (3)	C6—C7—C13—N12	64.2 (3)
N1—C2—C3—C4	−3.0 (4)	C8—C7—C13—N12	−58.2 (3)
C2—C3—C4—C5	0.9 (5)	O2—S1—C1'—C6'	−15.8 (3)
C3—C4—C5—C6	1.5 (5)	O3—S1—C1'—C6'	−146.5 (2)
C4—C5—C6—N1	−1.5 (4)	N12—S1—C1'—C6'	99.0 (2)
C4—C5—C6—C7	178.7 (3)	O2—S1—C1'—C2'	165.7 (2)
C2—N1—C6—C5	−0.8 (3)	O3—S1—C1'—C2'	35.0 (2)
C10—N1—C6—C5	177.8 (2)	N12—S1—C1'—C2'	−79.4 (2)
C2—N1—C6—C7	179.0 (2)	C6'—C1'—C2'—C3'	−1.3 (4)
C10—N1—C6—C7	−2.4 (3)	S1—C1'—C2'—C3'	177.2 (2)
C5—C6—C7—C8	−147.4 (2)	C1'—C2'—C3'—C4'	1.0 (4)
N1—C6—C7—C8	32.8 (3)	C2'—C3'—C4'—C5'	0.4 (5)
C5—C6—C7—C13	90.2 (3)	C2'—C3'—C4'—Cl1	−179.7 (2)
N1—C6—C7—C13	−89.6 (3)	C3'—C4'—C5'—C6'	−1.5 (4)
C6—C7—C8—C9	−62.1 (3)	Cl1—C4'—C5'—C6'	178.7 (2)
C13—C7—C8—C9	60.5 (3)	C4'—C5'—C6'—C1'	1.2 (4)
C7—C8—C9—C10	62.6 (3)	C2'—C1'—C6'—C5'	0.2 (4)
C7—C8—C9—C11	−61.9 (3)	S1—C1'—C6'—C5'	−178.2 (2)

### (1R,5R)-3-[(4-chlorophenyl)sulfonyl]-1,2,3,4,5,6-hexahydro-8H-1,5-methanopyrido[1,2-a][1,5]diazocin-8-one (II) Hydrogen-bond geometry (Å, º)

**Table:**

*D*—H···*A*	*D*—H	H···*A*	*D*···*A*	*D*—H···*A*
C5—H5*A*···O1^i^	0.93	2.61	3.375 (4)	140
C13—H13*B*···O1^i^	0.97	2.69	3.475 (3)	139
C5′—H5′*A*···O3^i^	0.93	2.42	3.198 (3)	142
C8—H8*A*···O3^ii^	0.97	2.56	3.424 (3)	149
C4—H4*A*···Cl1^iii^	0.93	2.94	3.764 (3)	148

Symmetry codes: (i) *x*−1, *y*, *z*; (ii) −*x*+1, *y*+1/2, −*z*+1/2; (iii) −*x*, *y*−1/2, −*z*+1/2.

### (1R,5R)-3-((3-nitrophenyl)sulfonyl)-1,2,3,4,5,6-hexahydro-8H-1,5-methanopyrido[1,2-a][1,5]diazocin-8-one (III) Crystal data

**Table:**

C_17_H_17_N_3_O_5_S	*F*(000) = 392
*M**_r_* = 375.39	*D*_x_ = 1.455 Mg m^−^^3^
Monoclinic, *P*2_1_	Cu *K*α radiation, λ = 1.54184 Å
*a* = 11.040 (2) Å	Cell parameters from 5501 reflections
*b* = 6.2621 (13) Å	θ = 4.0–71.4°
*c* = 12.424 (3) Å	µ = 2.00 mm^−^^1^
β = 94.03 (3)°	*T* = 296 K
*V* = 856.8 (3) Å^3^	Prizmatic, colorless
*Z* = 2	0.20 × 0.15 × 0.10 mm

### (1R,5R)-3-((3-nitrophenyl)sulfonyl)-1,2,3,4,5,6-hexahydro-8H-1,5-methanopyrido[1,2-a][1,5]diazocin-8-one (III) Data collection

**Table:**

XtaLAB Synergy, Single source at home/near, HyPix3000 diffractometer	2446 independent reflections
Radiation source: micro-focus sealed X-ray tube, PhotonJet (Cu) X-ray Source	2363 reflections with *I* > 2σ(*I*)
Detector resolution: 10.0000 pixels mm^-1^	*R*_int_ = 0.023
ω scans	θ_max_ = 71.5°, θ_min_ = 3.6°
Absorption correction: multi-scan (SADABS; Krause *et al.*, 2015)	*h* = −13→13
*T*_min_ = 0.639, *T*_max_ = 1.000	*k* = −6→7
7776 measured reflections	*l* = −15→15

### (1R,5R)-3-((3-nitrophenyl)sulfonyl)-1,2,3,4,5,6-hexahydro-8H-1,5-methanopyrido[1,2-a][1,5]diazocin-8-one (III) Refinement

**Table:**

Refinement on *F*^2^	Hydrogen site location: inferred from neighbouring sites
Least-squares matrix: full	H-atom parameters constrained
*R*[*F*^2^ > 2σ(*F*^2^)] = 0.039	*w* = 1/[σ^2^(*F*_o_^2^) + (0.0754*P*)^2^ + 0.1248*P*] where *P* = (*F*_o_^2^ + 2*F*_c_^2^)/3
*wR*(*F*^2^) = 0.114	(Δ/σ)_max_ < 0.001
*S* = 1.04	Δρ_max_ = 0.47 e Å^−^^3^
2446 reflections	Δρ_min_ = −0.22 e Å^−^^3^
235 parameters	Absolute structure: Flack *x* determined using 579 quotients [(*I*^+^)-(*I*^-^)]/[(*I*^+^)+(*I*^-^)] (Parsons *et al.*, 2013)
1 restraint	Absolute structure parameter: 0.017 (16)

### (1R,5R)-3-((3-nitrophenyl)sulfonyl)-1,2,3,4,5,6-hexahydro-8H-1,5-methanopyrido[1,2-a][1,5]diazocin-8-one (III) Special details

**Table:**

Geometry. All esds (except the esd in the dihedral angle between two l.s. planes) are estimated using the full covariance matrix. The cell esds are taken into account individually in the estimation of esds in distances, angles and torsion angles; correlations between esds in cell parameters are only used when they are defined by crystal symmetry. An approximate (isotropic) treatment of cell esds is used for estimating esds involving l.s. planes.

### (1R,5R)-3-((3-nitrophenyl)sulfonyl)-1,2,3,4,5,6-hexahydro-8H-1,5-methanopyrido[1,2-a][1,5]diazocin-8-one (III) Fractional atomic coordinates and isotropic or equivalent isotropic displacement parameters (Å^2^)

**Table:**

	*x*	*y*	*z*	*U*_iso_*/*U*_eq_	
S1	0.15419 (6)	0.10657 (14)	0.65911 (6)	0.0503 (2)	
O1	0.6728 (3)	−0.1378 (5)	0.7756 (3)	0.0777 (8)	
O2	0.1535 (2)	0.1620 (5)	0.54790 (18)	0.0696 (9)	
O3	0.1536 (3)	−0.1126 (4)	0.6914 (3)	0.0761 (8)	
O4	−0.1062 (4)	0.0114 (12)	0.9888 (4)	0.143 (2)	
O5	−0.2278 (4)	0.2805 (12)	0.9936 (3)	0.140 (2)	
N1	0.5589 (2)	0.1629 (4)	0.7462 (2)	0.0446 (6)	
N12	0.2750 (2)	0.2153 (4)	0.71985 (18)	0.0421 (5)	
N3	−0.1493 (3)	0.1778 (10)	0.9537 (3)	0.0916 (16)	
C2	0.6474 (3)	0.0176 (6)	0.7157 (3)	0.0557 (8)	
C3	0.7019 (3)	0.0648 (7)	0.6191 (3)	0.0639 (11)	
H3A	0.759057	−0.028879	0.594281	0.077*	
C4	0.6726 (3)	0.2440 (8)	0.5615 (3)	0.0638 (10)	
H4A	0.710971	0.272896	0.498892	0.077*	
C5	0.5843 (3)	0.3867 (6)	0.5960 (3)	0.0553 (8)	
H5A	0.564335	0.509117	0.556234	0.066*	
C6	0.5290 (3)	0.3440 (5)	0.6875 (2)	0.0436 (6)	
C7	0.4346 (3)	0.4905 (5)	0.7275 (3)	0.0492 (7)	
H7A	0.451784	0.635774	0.703421	0.059*	
C8	0.4401 (3)	0.4899 (7)	0.8499 (3)	0.0580 (9)	
H8A	0.520190	0.532007	0.879470	0.070*	
H8B	0.380876	0.588526	0.875750	0.070*	
C9	0.4120 (3)	0.2638 (7)	0.8834 (2)	0.0526 (8)	
H9A	0.413307	0.261073	0.962380	0.063*	
C10	0.5094 (3)	0.1127 (8)	0.8501 (2)	0.0539 (7)	
H10A	0.575597	0.113061	0.905801	0.065*	
H10B	0.476096	−0.030658	0.846320	0.065*	
C11	0.2850 (3)	0.1977 (6)	0.8394 (2)	0.0514 (8)	
H11A	0.269259	0.051691	0.860401	0.062*	
H11B	0.225028	0.289081	0.869484	0.062*	
C13	0.3064 (3)	0.4300 (6)	0.6827 (3)	0.0495 (7)	
H13A	0.248524	0.533033	0.706739	0.059*	
H13B	0.302369	0.432878	0.604459	0.059*	
C1'	0.0293 (3)	0.2314 (5)	0.7149 (2)	0.0449 (6)	
C2'	−0.0143 (3)	0.1467 (6)	0.8071 (2)	0.0517 (8)	
H2'A	0.014481	0.017681	0.835735	0.062*	
C3'	−0.1031 (3)	0.2624 (8)	0.8550 (3)	0.0606 (10)	
C4'	−0.1479 (3)	0.4520 (8)	0.8136 (4)	0.0693 (11)	
H4'A	−0.207272	0.525883	0.847895	0.083*	
C5'	−0.1040 (3)	0.5310 (7)	0.7212 (4)	0.0696 (10)	
H5'A	−0.135141	0.657808	0.691690	0.084*	
C6'	−0.0138 (3)	0.4237 (6)	0.6715 (3)	0.0558 (8)	
H6'A	0.017549	0.479393	0.609888	0.067*	

### (1R,5R)-3-((3-nitrophenyl)sulfonyl)-1,2,3,4,5,6-hexahydro-8H-1,5-methanopyrido[1,2-a][1,5]diazocin-8-one (III) Atomic displacement parameters (Å^2^)

**Table:**

	*U*^11^	*U*^22^	*U*^33^	*U*^12^	*U*^13^	*U*^23^
S1	0.0495 (4)	0.0415 (4)	0.0608 (4)	0.0046 (3)	0.0094 (3)	−0.0112 (4)
O1	0.0648 (16)	0.0635 (19)	0.105 (2)	0.0234 (14)	0.0084 (14)	0.0117 (17)
O2	0.0688 (14)	0.090 (3)	0.0508 (11)	−0.0015 (14)	0.0079 (10)	−0.0178 (13)
O3	0.0725 (17)	0.0361 (14)	0.121 (2)	0.0036 (12)	0.0151 (16)	−0.0143 (15)
O4	0.109 (3)	0.205 (6)	0.120 (3)	0.006 (4)	0.038 (2)	0.085 (4)
O5	0.123 (3)	0.186 (6)	0.120 (3)	−0.016 (4)	0.071 (3)	−0.029 (4)
N1	0.0405 (11)	0.0402 (15)	0.0533 (12)	0.0025 (10)	0.0038 (9)	−0.0029 (11)
N12	0.0437 (12)	0.0376 (14)	0.0458 (11)	0.0058 (11)	0.0079 (9)	0.0014 (10)
N3	0.060 (2)	0.148 (5)	0.069 (2)	−0.013 (2)	0.0244 (16)	0.009 (3)
C2	0.0444 (15)	0.0499 (19)	0.072 (2)	0.0058 (15)	0.0022 (14)	−0.0110 (18)
C3	0.0494 (16)	0.069 (3)	0.074 (2)	0.0040 (17)	0.0143 (15)	−0.022 (2)
C4	0.060 (2)	0.072 (3)	0.0609 (18)	−0.0118 (19)	0.0194 (16)	−0.0169 (19)
C5	0.0612 (19)	0.056 (2)	0.0495 (16)	−0.0082 (16)	0.0095 (13)	−0.0003 (15)
C6	0.0436 (14)	0.0395 (16)	0.0477 (14)	−0.0042 (12)	0.0023 (11)	−0.0048 (13)
C7	0.0570 (16)	0.0318 (16)	0.0595 (17)	0.0019 (13)	0.0089 (13)	−0.0002 (13)
C8	0.0631 (18)	0.054 (2)	0.0570 (17)	0.0011 (17)	0.0084 (14)	−0.0178 (16)
C9	0.0546 (17)	0.062 (2)	0.0415 (13)	0.0025 (16)	0.0069 (12)	−0.0016 (15)
C10	0.0491 (14)	0.0588 (19)	0.0539 (15)	0.0031 (18)	0.0039 (12)	0.0138 (18)
C11	0.0500 (15)	0.058 (2)	0.0474 (14)	0.0080 (15)	0.0120 (12)	0.0053 (15)
C13	0.0505 (16)	0.0400 (17)	0.0578 (16)	0.0086 (14)	0.0039 (13)	0.0096 (15)
C1'	0.0380 (13)	0.0422 (17)	0.0542 (15)	0.0021 (12)	0.0018 (11)	−0.0016 (14)
C2'	0.0394 (13)	0.055 (2)	0.0613 (16)	−0.0045 (14)	0.0056 (12)	0.0088 (16)
C3'	0.0426 (15)	0.083 (3)	0.0572 (17)	−0.0108 (17)	0.0072 (13)	−0.0053 (19)
C4'	0.0446 (16)	0.076 (3)	0.089 (3)	0.0053 (18)	0.0116 (16)	−0.024 (2)
C5'	0.0525 (17)	0.056 (2)	0.100 (3)	0.0152 (17)	0.0041 (18)	0.004 (2)
C6'	0.0490 (16)	0.051 (2)	0.0682 (19)	0.0059 (15)	0.0058 (14)	0.0066 (17)

### (1R,5R)-3-((3-nitrophenyl)sulfonyl)-1,2,3,4,5,6-hexahydro-8H-1,5-methanopyrido[1,2-a][1,5]diazocin-8-one (III) Geometric parameters (Å, º)

**Table:**

S1—O2	1.424 (3)	C7—H7A	0.9800
S1—O3	1.430 (3)	C8—C9	1.514 (6)
S1—N12	1.634 (3)	C8—H8A	0.9700
S1—C1'	1.768 (3)	C8—H8B	0.9700
O1—C2	1.245 (5)	C9—C10	1.512 (5)
O4—N3	1.214 (8)	C9—C11	1.526 (5)
O5—N3	1.212 (7)	C9—H9A	0.9800
N1—C6	1.376 (4)	C10—H10A	0.9700
N1—C2	1.407 (4)	C10—H10B	0.9700
N1—C10	1.471 (4)	C11—H11A	0.9700
N12—C13	1.471 (4)	C11—H11B	0.9700
N12—C11	1.485 (4)	C13—H13A	0.9700
N3—C3'	1.460 (5)	C13—H13B	0.9700
C2—C3	1.411 (5)	C1'—C2'	1.379 (4)
C3—C4	1.357 (6)	C1'—C6'	1.390 (5)
C3—H3A	0.9300	C2'—C3'	1.386 (5)
C4—C5	1.411 (6)	C2'—H2'A	0.9300
C4—H4A	0.9300	C3'—C4'	1.372 (7)
C5—C6	1.354 (4)	C4'—C5'	1.370 (6)
C5—H5A	0.9300	C4'—H4'A	0.9300
C6—C7	1.499 (4)	C5'—C6'	1.383 (5)
C7—C8	1.519 (4)	C5'—H5'A	0.9300
C7—C13	1.531 (5)	C6'—H6'A	0.9300
			
O2—S1—O3	120.4 (2)	C10—C9—C8	110.3 (3)
O2—S1—N12	107.14 (14)	C10—C9—C11	112.7 (3)
O3—S1—N12	106.87 (16)	C8—C9—C11	110.8 (3)
O2—S1—C1'	108.78 (16)	C10—C9—H9A	107.6
O3—S1—C1'	107.17 (17)	C8—C9—H9A	107.6
N12—S1—C1'	105.56 (13)	C11—C9—H9A	107.6
C6—N1—C2	122.4 (3)	N1—C10—C9	114.9 (3)
C6—N1—C10	123.5 (3)	N1—C10—H10A	108.5
C2—N1—C10	114.0 (3)	C9—C10—H10A	108.5
C13—N12—C11	112.2 (3)	N1—C10—H10B	108.5
C13—N12—S1	116.0 (2)	C9—C10—H10B	108.5
C11—N12—S1	115.6 (2)	H10A—C10—H10B	107.5
O5—N3—O4	125.6 (5)	N12—C11—C9	109.9 (2)
O5—N3—C3'	116.9 (6)	N12—C11—H11A	109.7
O4—N3—C3'	117.5 (4)	C9—C11—H11A	109.7
O1—C2—N1	118.4 (3)	N12—C11—H11B	109.7
O1—C2—C3	125.5 (3)	C9—C11—H11B	109.7
N1—C2—C3	116.1 (3)	H11A—C11—H11B	108.2
C4—C3—C2	121.4 (3)	N12—C13—C7	110.2 (2)
C4—C3—H3A	119.3	N12—C13—H13A	109.6
C2—C3—H3A	119.3	C7—C13—H13A	109.6
C3—C4—C5	120.5 (3)	N12—C13—H13B	109.6
C3—C4—H4A	119.7	C7—C13—H13B	109.6
C5—C4—H4A	119.7	H13A—C13—H13B	108.1
C6—C5—C4	119.4 (4)	C2'—C1'—C6'	121.7 (3)
C6—C5—H5A	120.3	C2'—C1'—S1	118.9 (3)
C4—C5—H5A	120.3	C6'—C1'—S1	119.1 (2)
C5—C6—N1	120.1 (3)	C1'—C2'—C3'	117.0 (3)
C5—C6—C7	121.5 (3)	C1'—C2'—H2'A	121.5
N1—C6—C7	118.4 (3)	C3'—C2'—H2'A	121.5
C6—C7—C8	110.5 (3)	C4'—C3'—C2'	122.5 (3)
C6—C7—C13	112.0 (3)	C4'—C3'—N3	119.4 (4)
C8—C7—C13	109.5 (3)	C2'—C3'—N3	118.1 (4)
C6—C7—H7A	108.2	C5'—C4'—C3'	119.2 (3)
C8—C7—H7A	108.2	C5'—C4'—H4'A	120.4
C13—C7—H7A	108.2	C3'—C4'—H4'A	120.4
C9—C8—C7	106.5 (3)	C4'—C5'—C6'	120.5 (4)
C9—C8—H8A	110.4	C4'—C5'—H5'A	119.8
C7—C8—H8A	110.4	C6'—C5'—H5'A	119.8
C9—C8—H8B	110.4	C5'—C6'—C1'	119.1 (3)
C7—C8—H8B	110.4	C5'—C6'—H6'A	120.5
H8A—C8—H8B	108.6	C1'—C6'—H6'A	120.5
			
O2—S1—N12—C13	38.6 (2)	C8—C9—C10—N1	−36.5 (4)
O3—S1—N12—C13	168.9 (2)	C11—C9—C10—N1	87.9 (4)
C1'—S1—N12—C13	−77.3 (2)	C13—N12—C11—C9	−55.3 (4)
O2—S1—N12—C11	173.1 (2)	S1—N12—C11—C9	168.5 (2)
O3—S1—N12—C11	−56.6 (3)	C10—C9—C11—N12	−65.9 (4)
C1'—S1—N12—C11	57.3 (3)	C8—C9—C11—N12	58.2 (4)
C6—N1—C2—O1	177.1 (3)	C11—N12—C13—C7	56.7 (3)
C10—N1—C2—O1	1.8 (5)	S1—N12—C13—C7	−167.3 (2)
C6—N1—C2—C3	−1.8 (4)	C6—C7—C13—N12	62.7 (3)
C10—N1—C2—C3	−177.0 (3)	C8—C7—C13—N12	−60.3 (4)
O1—C2—C3—C4	−176.7 (4)	O2—S1—C1'—C2'	157.4 (3)
N1—C2—C3—C4	2.0 (5)	O3—S1—C1'—C2'	25.8 (3)
C2—C3—C4—C5	−1.3 (6)	N12—S1—C1'—C2'	−87.9 (3)
C3—C4—C5—C6	0.2 (5)	O2—S1—C1'—C6'	−29.2 (3)
C4—C5—C6—N1	0.1 (5)	O3—S1—C1'—C6'	−160.8 (3)
C4—C5—C6—C7	−180.0 (3)	N12—S1—C1'—C6'	85.5 (3)
C2—N1—C6—C5	0.8 (4)	C6'—C1'—C2'—C3'	−0.3 (5)
C10—N1—C6—C5	175.6 (3)	S1—C1'—C2'—C3'	173.0 (2)
C2—N1—C6—C7	−179.2 (3)	C1'—C2'—C3'—C4'	0.7 (5)
C10—N1—C6—C7	−4.4 (4)	C1'—C2'—C3'—N3	−179.0 (3)
C5—C6—C7—C8	−147.0 (3)	O5—N3—C3'—C4'	1.1 (6)
N1—C6—C7—C8	33.0 (4)	O4—N3—C3'—C4'	−178.9 (5)
C5—C6—C7—C13	90.6 (4)	O5—N3—C3'—C2'	−179.3 (4)
N1—C6—C7—C13	−89.4 (3)	O4—N3—C3'—C2'	0.8 (6)
C6—C7—C8—C9	−62.1 (3)	C2'—C3'—C4'—C5'	0.2 (6)
C13—C7—C8—C9	61.7 (4)	N3—C3'—C4'—C5'	179.8 (4)
C7—C8—C9—C10	64.2 (3)	C3'—C4'—C5'—C6'	−1.4 (6)
C7—C8—C9—C11	−61.3 (3)	C4'—C5'—C6'—C1'	1.8 (6)
C6—N1—C10—C9	6.1 (5)	C2'—C1'—C6'—C5'	−0.9 (5)
C2—N1—C10—C9	−178.7 (3)	S1—C1'—C6'—C5'	−174.1 (3)

### (1R,5R)-3-((3-nitrophenyl)sulfonyl)-1,2,3,4,5,6-hexahydro-8H-1,5-methanopyrido[1,2-a][1,5]diazocin-8-one (III) Hydrogen-bond geometry (Å, º)

**Table:**

*D*—H···*A*	*D*—H	H···*A*	*D*···*A*	*D*—H···*A*
C4′—H4′*A*···O1^i^	0.93	2.61	3.257 (5)	127
C10—H10*A*···O5^ii^	0.97	2.58	3.460 (6)	151
C11—H11*A*···O5^iii^	0.97	2.55	3.428 (7)	150
C13—H13*A*···O3^iv^	0.97	2.46	3.330 (4)	150

Symmetry codes: (i) *x*−1, *y*+1, *z*; (ii) *x*+1, *y*, *z*; (iii) −*x*, *y*−1/2, −*z*+2; (iv) *x*, *y*+1, *z*.
